# Natural Bioactive Compounds Targeting Gut Barrier Integrity and Metabolic Endotoxemia in Cardiometabolic Disease: Mechanistic Insights and Translational Perspectives

**DOI:** 10.3390/molecules31111840

**Published:** 2026-05-27

**Authors:** Roko Šantić, Lovre Martinović, Nikola Pavlović, Dinko Martinović, Josip Vrdoljak, Marko Kumrić, Marino Vilović, Joško Božić

**Affiliations:** 1 Department of Pathophysiology, University of Split School of Medicine, 21000 Split, Croatia; roko.santic@mefst.hr (R.Š.); lovre.martinovic@mefst.hr (L.M.); nikola.pavlovic@mefst.hr (N.P.); josip.vrdoljak@mefst.hr (J.V.); marko.kumric@mefst.hr (M.K.); marino.vilovic@mefst.hr (M.V.); 2 Laboratory for Cardiometabolic Research, University of Split School of Medicine, 21000 Split, Croatia; 3 Department of Maxillofacial Surgery, University Hospital of Split, 21000 Split, Croatia; dmartinovic@kbsplit.hr

**Keywords:** intestinal barrier dysfunction, metabolic endotoxemia, cardiometabolic diseases, chronic low-grade inflammation, natural bioactive compounds

## Abstract

Cardiometabolic diseases are increasingly recognized as disorders of chronic low-grade systemic inflammation and gut barrier dysfunction that mutually reinforce one another. Each condition amplifies the other through progressive injury to the intestinal epithelium. Compromise of the mucus layer, altered tight junction dynamics, dysbiosis, and impaired epithelial restitution promote intestinal permeability and enable the translocation of lipopolysaccharide and other microbial products into the circulation, thereby inducing metabolic endotoxemia. This gut derived inflammatory signal activates Toll like receptor 4, nuclear factor kappa B, and inflammasome associated pathways, linking barrier dysfunction to insulin resistance, hepatic steatosis, adipose tissue inflammation, endothelial activation, and vascular injury. Here, we examine the gut barrier as an immunometabolic interface and synthesize current evidence connecting its disruption to endotoxin driven cardiometabolic pathology. We further evaluate selected natural bioactive compounds, including curcumin, resveratrol, quercetin, epigallocatechin gallate, berberine, anthocyanins, omega 3 polyunsaturated fatty acids, and dietary polysaccharides, as gut targeted interventions capable of reinforcing junctional integrity, restoring mucus and microbial homeostasis, lowering endotoxin burden, and attenuating inflammatory signaling. Finally, we highlight the principal translational barriers that currently limit clinical implementation, including pharmacokinetic variability, microbiota dependent biotransformation, source standardization, and the lack of robust, standardized biomarkers of barrier restoration and metabolic endotoxemia.

## 1. Literature Search and Evidence Selection

This review was based on a narrative literature search designed to identify experimental, clinical, and translational evidence linking natural products with gut barrier integrity, gut microbiota, low-grade inflammation, and metabolic endotoxemia. Searches were conducted in PubMed, Scopus, Web of Science, and Google Scholar using combinations of terms related to the gut barrier (“intestinal permeability”, “tight junctions”, “mucus layer”, “epithelial barrier”, “zonulin”), metabolic endotoxemia (“lipopolysaccharide”, “LPS”, “endotoxin”, “TLR4”, “low-grade inflammation”), gut microbiota (“dysbiosis”, “short-chain fatty acids”, “bile acids”, “microbial metabolites”), and selected bioactive compounds or compound classes, including polyphenols, curcumin, resveratrol, quercetin, epigallocatechin gallate, anthocyanins, berberine, and other plant-derived metabolites. Reference lists of relevant reviews and primary studies were additionally screened to identify articles not captured by database searches.

Priority was given to systematic reviews, meta-analyses, randomized controlled trials, prospective cohort studies, and well-characterized animal or cell-culture studies that directly assessed barrier function, microbiota composition, inflammatory signaling, or metabolic outcomes. Mechanistic studies were included when they provided biologically plausible links between compound exposure and pathways relevant to epithelial integrity, such as AMPK, NF-κB, MAPK, Nrf2, tight-junction regulation, oxidative stress, or immune-cell activation. Studies were excluded when they were not available in English, lacked relevance to the gut barrier or metabolic endotoxemia axis, reported only nonspecific antioxidant effects without mechanistic or physiological endpoints, or were based solely on unsupported secondary citations. Because this article is a narrative review rather than a systematic review, formal risk-of-bias assessment and quantitative evidence synthesis were not performed.

## 2. Introduction

Cardiometabolic diseases, encompassing type 2 diabetes mellitus (T2DM), atherosclerotic cardiovascular disease (CVD), non-alcoholic fatty liver disease (NAFLD), and metabolic syndrome, represent the dominant cause of morbidity and mortality worldwide and share a common pathophysiological substrate of chronic, low-grade systemic inflammation [1]. The global burden is substantial: an estimated 537 million adults were living with diabetes in 2021, with projections indicating 783 million by 2045 [2]; CVD accounted for approximately 17.9 million deaths annually as of 2019, representing 32% of all global mortality [3]; and NAFLD affects an estimated 32% of the adult population worldwide, with prevalence increasing over recent decades [4]. Over the past two decades, accumulating evidence has repositioned the gastrointestinal tract from a passive absorptive organ to an immunologically active barrier whose functional state directly influences systemic inflammatory tone and metabolic homeostasis [5]. Central to this reconceptualization is the observation that structural and functional compromise of the intestinal epithelial barrier permits the translocation of microbial products, most notably lipopolysaccharide (LPS), from the gut lumen into the portal and systemic circulation, a phenomenon termed metabolic endotoxemia [6].

Metabolic endotoxemia initiates and sustains a signaling cascade through Toll-like receptor 4 (TLR4) and downstream nuclear factor-kappa B (NF-κB) pathways that drives macrophage activation, pro-inflammatory cytokine release, insulin resistance, hepatic steatosis, and endothelial dysfunction, thereby linking intestinal permeability to the full spectrum of cardiometabolic pathology [7]. Conventional pharmacological interventions target downstream manifestations of this inflammatory cascade, yet they do not address the upstream breach in barrier integrity that initiates and perpetuates it [8].

**Figure 1:**
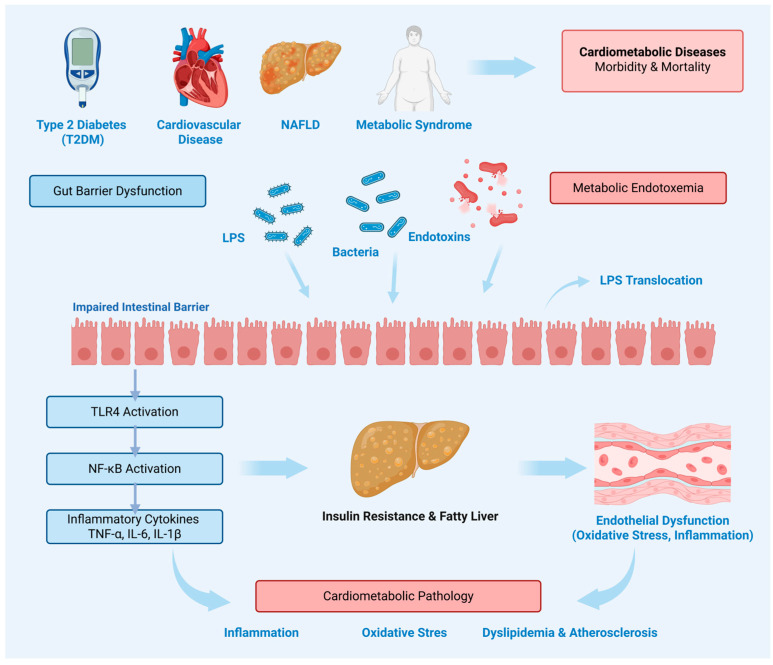
This schematic illustrates the role of gut barrier dysfunction in the development of cardiometabolic diseases. Impairment of the mucus layer, epithelial tight junctions, and immune barrier function increases intestinal permeability and enables the translocation of lipopolysaccharide (LPS), a Gram-negative bacterial cell-wall component, into the portal and systemic circulation, leading to metabolic endotoxemia. LPS activates Toll-like receptor 4 (TLR4) and downstream NF-κB signaling, promoting the release of pro-inflammatory cytokines such as TNF-α, IL-6, and IL-1β. These mechanisms are linked to the four disease categories discussed in this review: in type 2 diabetes mellitus, LPS-driven inflammation aggravates insulin resistance and β-cell dysfunction; in non-alcoholic fatty liver disease (NAFLD), portal LPS exposure promotes Kupffer-cell activation, hepatic steatosis, and inflammatory progression; in atherosclerotic cardiovascular disease, circulating LPS contributes to endothelial activation, monocyte recruitment, and plaque destabilization; and in metabolic syndrome, endotoxemia-related inflammation connects central obesity, dyslipidemia, hepatic insulin resistance, and systemic metabolic dysfunction. Created in BioRender. Kumric, M. (2026) https://BioRender.com/3vjws3l (accessed on 21 May 2026).

Natural bioactive compounds, including polyphenols, alkaloids, polyunsaturated fatty acids, and dietary polysaccharides, have attracted attention as candidate gut-targeted interventions because many of them reach the intestinal mucosa at pharmacologically relevant concentrations regardless of systemic bioavailability and exert barrier-protective, anti-inflammatory, and microbiota-modulatory effects through well-characterized molecular mechanisms [9,10,11]. This review examines the structural and functional organization of the gut barrier, delineates the pathophysiology of barrier dysfunction and metabolic endotoxemia, evaluates the mechanistic basis and translational evidence for selected natural compounds that target intestinal permeability and endotoxin-driven inflammation, and identifies the critical gaps that must be resolved before these compounds can transition from preclinical promise to standardized clinical application in cardiometabolic disease. The proposed disease-specific mechanisms linking gut barrier dysfunction, LPS-driven metabolic endotoxemia, and major cardiometabolic diseases are summarized in Figure 1.

## 3. Gut Barrier: Structure and Role

The gut barrier is a highly specialized interface that reconciles two seemingly opposing tasks: it must permit efficient absorption of nutrients, water and electrolytes while at the same time limiting host exposure to the dense microbial and antigenic load in the gut lumen [12,13]. Structurally, it is organized as a multilayered system composed of an inner epithelial monolayer with its junctional complexes, an overlying mucus and antimicrobial shield, and an underlying immune compartment in the lamina propria [12,14]. This organization allows fine-tuned control of permeability so that physiological flux of nutrients and selected luminal signals is maintained without excessive translocation of microbes and their products into the circulation [13,15].

At the core of the gut barrier lies a single layer of polarized intestinal epithelial cells [12,16,17]. This epithelium is composed of several specialized lineages, including absorptive enterocytes, mucus-secreting goblet cells, antimicrobial peptide-producing Paneth cells and hormone-secreting enteroendocrine cells, all continuously replenished from Lgr5^+^ stem cells located in the crypts [12,16,17]. High turnover and tightly regulated differentiation are essential to maintain an intact and resilient surface that can rapidly repair microscopic injury caused by dietary, chemical and mechanical stressors [12,17]. In the context of cardiometabolic disease, alterations in epithelial renewal and stress responses can lower the threshold at which environmental or metabolic insults translate into barrier dysfunction and low-grade inflammation [18,19,20].

Adjacent epithelial cells are tightly connected by a junctional complex that determines paracellular permeability [12,13]. Tight junctions form the apical-most component and comprise transmembrane proteins such as claudins, occludin and junctional adhesion molecules (JAMs), linked to the perijunctional actin cytoskeleton via scaffold proteins including zonula occludens-1 (ZO-1) and ZO-2 [12,15,21]. By dynamically regulating the composition and phosphorylation state of these proteins, epithelial cells can adapt paracellular flux to physiological needs, whereas metabolic, inflammatory or microbial stress can drive junctional disassembly and increased leakiness [15,21,22]. Beneath tight junctions, adherens junctions and desmosomes provide mechanical cohesion and stabilize cell–cell contacts, so that disruption of any component of this junctional apparatus can compromise barrier integrity [12,15,21].

The epithelial surface is covered by a mucus layer rich in heavily glycosylated mucins, predominantly MUC2, secreted by goblet cells [14,23]. This hydrated gel physically separates luminal microbiota from epithelial cells, creating a gradient in microbial density and endotoxin concentration from the lumen toward the epithelial surface [14,23,24]. Within this matrix, antimicrobial peptides such as defensins, cathelicidins and C-type lectins (e.g., RegIII family) produced by Paneth cells and other epithelial subsets selectively kill or repel bacteria approaching the epithelium, providing a form of localized, “immunologically silent” microbial control [15,22,24]. Secretory IgA produced by lamina propria plasma cells and transported across the epithelium into the mucus further contributes by coating bacteria and toxins, promoting immune exclusion and maintaining a non-inflammatory relationship with commensal microbes [22,24].

Beyond its structural components, the gut barrier is increasingly recognized as a central immunometabolic platform [13,18,19,20,25]. Intestinal epithelial cells express pattern-recognition receptors and nutrient-sensing pathways, integrate signals from microbial metabolites such as short-chain fatty acids, secondary bile acids and tryptophan-derived indoles, and translate these cues into changes in junctional integrity, mucus production and cytokine output [26,27,28]. Through these mechanisms, the gut barrier not only separates but also actively interprets luminal signals, thereby shaping systemic inflammatory tone and metabolic homeostasis [18,20,27]. This dual role—as both physical barrier and signaling hub—provides the conceptual basis for understanding how subtle alterations in barrier structure and function can contribute to metabolic endotoxemia and cardiometabolic inflammation, which are addressed in subsequent sections of this review [19,20,26].

## 4. Pathophysiology of Barrier Dysfunction

Gut barrier dysfunction refers to a state of impaired intestinal integrity characterized by increased permeability, disruption of the epithelial and mucus barriers, and loss of junctional control, resulting in enhanced translocation of luminal microbial products and other harmful molecules [13,29].

Multiple metabolic and dietary stressors contribute to this process. Among them, a Western-style or high-fat diet is one of the best-established triggers, as it promotes shifts in gut microbial composition, enhances luminal inflammatory pressure, and increases intestinal permeability [30,31]. Obesity further amplifies barrier disruption through chronic low-grade inflammation, adipose-derived inflammatory mediators, and metabolic stress, while hyperglycemia can directly impair epithelial function by altering junctional integrity and epithelial homeostasis [32,33,34]. Lipotoxicity and oxidative stress add another layer of injury, as excess fatty acids and reactive oxygen species damage epithelial cells and weaken barrier resilience [35,36,37,38]. In parallel, dysbiosis reduces beneficial microbial metabolites and increases exposure to microbiota-derived injury signals, thereby sustaining mucosal immune activation [31,39]. Together, these factors act in a mutually reinforcing manner, driving persistent barrier dysfunction rather than functioning as isolated insults.

At the molecular and cellular level, barrier impairment is primarily expressed through disruption of intercellular junctions, epithelial damage, and defective mucosal maintenance [15,29,40]. A key event is the altered expression and redistribution of tight junction proteins, including claudins, occludin, and ZO-1, which weakens paracellular sealing and destabilizes epithelial cohesion [28,41,42]. This is frequently accompanied by epithelial stress, mitochondrial dysfunction, and apoptosis, all of which reduce the capacity of the mucosa to preserve an intact absorptive surface [40,43,44]. In parallel, depletion or disorganization of the MUC2-based mucus layer diminishes the physical separation between luminal microbes and epithelial cells, thereby increasing susceptibility to direct microbial contact [45,46,47]. Barrier integrity is further compromised when epithelial renewal is impaired, as defective stem-cell-driven restitution limits repair of injured surfaces [48,49]. Finally, immune dysregulation and microbiota-derived injury signals sustain local cytokine release, epithelial stress, and inflammatory activation, converting transient epithelial injury into persistent barrier failure [50,51].

The functional consequence of these molecular and cellular alterations is increased intestinal permeability and progressive loss of barrier integrity. Paracellular transport represents a key pathway of increased intestinal permeability and occurs through tight junctions between adjacent epithelial cells [52,53]. Two functionally distinct routes can be distinguished: the pore pathway and the leak pathway. The pore pathway is regulated by claudin proteins, which form size and charge-selective channels that primarily permit the passage of small ions and water. In contrast, the leak pathway allows the transit of larger molecules and is particularly enhanced under inflammatory conditions [54]. This pathway is regulated by MLCK1-dependent cytoskeletal contraction and occludin endocytosis, processes that are further amplified by pro-inflammatory cytokines such as TNF and IL-1β [52,55].

Besides paracellular leakage, barrier dysfunction may also involve abnormalities in transcellular passage across the intestinal epithelium. In physiological conditions, the amount of intact luminal protein transported through enterocytes is minimal, highlighting the normally high efficiency of epithelial barrier control [56,57]. During inflammation, however, this route can become upregulated. Pro-inflammatory cytokines such as TNF-α have been shown to increase intracellular uptake of luminal antigens, while IFN-γ further facilitates the epithelial transfer of larger macromolecules [56,58]. In addition, a distinct epithelial subpopulation, referred to as RACE cells (rapid antigen uptake into the cytosol enterocytes), appears particularly capable of antigen translocation [59,60]. These cells are subsequently shed into the intestinal lumen, creating focal epithelial discontinuities, and are thought to represent an early stage of apoptosis [61,62]. Collectively, these findings suggest that altered transcellular transport not only increases luminal antigen passage, but may also directly contribute to structural weakening of the epithelial barrier.

Importantly, loss of barrier integrity cannot be reduced to increased molecular flux alone, but also includes impaired epithelial resilience. A stressed epithelium is less able to preserve structural continuity, maintain functional homeostasis, and undergo efficient restitution following injury [29,49]. Consequently, transient epithelial disturbances are more likely to evolve into sustained mucosal damage and persistent barrier dysfunction.

## 5. Metabolic Endotoxemia and Signaling

The term metabolic endotoxemia denotes a state of chronically elevated circulating lipopolysaccharide (LPS), typically at plasma concentrations in the range of 10–50 pg/mL as measured by the Limulus amebocyte lysate (LAL) assay, approximately 10–50-fold lower than the levels observed in clinical sepsis (median ~300 pg/mL), arising not from acute infection but from the continuous low-level translocation of Gram-negative bacterial cell-wall components across a compromised gut barrier [59,63,64]. Unlike septic endotoxemia, which triggers fulminant systemic inflammation, metabolic endotoxemia produces a subclinical inflammatory state that is sustained over months to years, a temporal profile that aligns with the slow progression of cardiometabolic disease [6,64,65]. The principal source of this LPS is the commensal Gram-negative microbiota of the distal ileum and colon, where barrier dysfunction, as outlined in the preceding section, permits paracellular and transcellular translocation of endotoxin into the portal venous system and, subsequently, into the systemic circulation [6]. The lactulose/mannitol ratio test remains the most widely used functional assessment of small intestinal permeability in clinical settings [66].

Once translocated across the epithelium, LPS enters the portal blood bound to LPS-binding protein (LBP), a hepatic acute-phase glycoprotein that catalyzes the transfer of LPS monomers to the co-receptor CD14 on the surface of innate immune cells [67,68]. The LPS–CD14 complex is then delivered to the signaling receptor Toll-like receptor 4 (TLR4), whose co-receptor MD-2 provides the hydrophobic pocket that accommodates the lipid A moiety of LPS, completing receptor dimerization and initiating signal transduction [69]. This recognition step is the molecular event that converts a passive permeability defect into an active inflammatory cascade.

TLR4 dimerization engages two parallel intracellular signaling arms. The MyD88-dependent pathway, activated at the plasma membrane, recruits the adaptor proteins TIRAP and MyD88, leading to sequential activation of IRAK4, IRAK1, and TRAF6, which in turn activates the TAK1 kinase complex [70]. TAK1 phosphorylates the IκB kinase (IKK) complex, triggering proteasomal degradation of IκBα and nuclear translocation of NF-κB p50/p65 heterodimers, which drive transcription of TNF-α, IL-6, IL-1β, cyclooxygenase-2, and inducible nitric oxide synthase [71,72]. In parallel, TAK1 activates the MAPK cascade through JNK, ERK1/2, and p38, amplifying cytokine output and engaging AP-1-dependent transcriptional programs [72,73]. The TRIF-dependent pathway, initiated following TLR4 endosomal internalization, signals through TRAM and TRIF to activate IRF3 and a delayed phase of NF-κB activation, promoting type I interferon production and sustained inflammatory signaling that persists beyond the initial LPS encounter [74].

A critical amplification node within this cascade is the NLRP3 inflammasome. LPS-driven NF-κB transcription of pro-IL-1β and NLRP3 constitutes the priming signal, while secondary danger-associated molecular patterns, mitochondrial reactive oxygen species, or extracellular ATP provide the activation signal that triggers NLRP3 oligomerization, ASC speck formation, and caspase-1-dependent cleavage of pro-IL-1β and pro-IL-18 into their bioactive secreted forms [75,76]. In parallel, caspase-1-mediated cleavage of gasdermin D generates membrane pores that mediate pyroptotic cell death, further releasing intracellular alarmins and perpetuating the inflammatory cycle [77]. The resulting IL-1β and IL-18 act on both local and distant tissues, reinforcing insulin resistance in adipocytes, promoting hepatocyte lipogenesis, and driving endothelial activation [76].

Metabolic endotoxemia also engages non-canonical inflammatory pathways. In hepatocytes and adipocytes, LPS-activated TLR4 signaling induces ceramide biosynthesis through upregulation of serine palmitoyltransferase, generating lipotoxic intermediates that independently impair insulin receptor substrate-1 (IRS-1) phosphorylation and promote ER stress [78]. Simultaneously, LPS stimulates fetuin-A release from the liver, which acts as an endogenous TLR4 ligand on adipocytes and macrophages, creating a hepato-adipose amplification loop that sustains inflammation even in the absence of continued endotoxin influx [79].

## 6. Relevance in Cardiometabolic Disease

The signaling cascades initiated by metabolic endotoxemia converge on the principal tissue compartments that define cardiometabolic disease: adipose tissue, the liver, the vascular endothelium, and the pancreatic islet. Although these pathways are mechanistically interconnected, their disease-specific expression differs across the four cardiometabolic categories emphasized in this review. In T2DM, LPS-driven inflammation promotes adipose and hepatic insulin resistance and contributes to β-cell dysfunction; in NAFLD, portal LPS exposure activates Kupffer-cell inflammatory signaling and supports steatosis and fibrogenic progression; in atherosclerotic CVD, circulating LPS and endotoxemia-related markers promote endothelial activation and plaque vulnerability; and in metabolic syndrome, the same endotoxemia-driven inflammatory axis integrates central obesity, dyslipidemia, hepatic insulin resistance, and systemic metabolic dysfunction [6,80,81,82]. In each compartment, chronic LPS–TLR4 activation drives distinct but interconnected pathological processes that collectively account for the clustering of insulin resistance, dyslipidemia, hepatic steatosis, hypertension, and accelerated atherosclerosis within the metabolic syndrome phenotype [80,81,82].

In adipose tissue, LPS activates resident and recruited macrophages through TLR4, shifting their polarization from the anti-inflammatory M2 phenotype toward the classically activated M1 state [83,84]. M1 macrophages release TNF-α, IL-6, and IL-1β into the adipose microenvironment, where TNF-α phosphorylates IRS-1 at inhibitory serine residues via JNK, directly impairing insulin-stimulated GLUT4 translocation and adipocyte glucose uptake [85,86]. Simultaneously, NF-κB-driven suppression of adiponectin transcription removes a key insulin-sensitizing adipokine from systemic circulation, while LPS-stimulated ceramide accumulation further antagonizes Akt/PKB signaling downstream of the insulin receptor [87,88,89,90]. The net result is adipose insulin resistance accompanied by unrestrained lipolysis, increasing circulating free fatty acids and providing substrate for ectopic lipid deposition in liver and vasculature [87,88,91].

The liver occupies an anatomically exposed position in the endotoxemia–cardiometabolic axis because it receives the entirety of the portal venous blood and its associated LPS load before any systemic dilution [92]. Kupffer cells, the hepatic resident macrophages, are activated by portal LPS through TLR4/NF-κB, releasing TNF-α and IL-1β that act on adjacent hepatocytes to upregulate de novo lipogenesis via SREBP-1c while simultaneously suppressing fatty acid β-oxidation through inhibition of PPARα [92,93,94]. This metabolic reprogramming produces hepatic steatosis, the hallmark of NAFLD, and when sustained, progresses to steatohepatitis through lipotoxicity-driven hepatocyte injury, stellate cell activation, and fibrogenesis [94,95]. Hepatic insulin resistance further amplifies systemic metabolic dysfunction by impairing glycogen synthesis and de-repressing gluconeogenesis, contributing to fasting hyperglycemia, while impaired hepatic VLDL metabolism drives the atherogenic dyslipidemia characteristic of the metabolic syndrome [96,97].

At the vascular level, circulating LPS and pro-inflammatory cytokines converge on the endothelium to initiate and accelerate atherogenesis. LPS directly activates endothelial TLR4, upregulating VCAM-1, ICAM-1, and E-selectin, which recruit circulating monocytes into the subendothelial space where they differentiate into foam cells [98]. Within established plaques, TLR4 activation on intraplaque macrophages enhances MMP-2 and MMP-9 secretion and necrotic core expansion in murine and ex vivo human tissue models, promoting the vulnerable plaque phenotype associated with acute coronary events [99,100]. Elevated plasma LBP and soluble CD14 independently predict incident cardiovascular events and correlate with carotid intima-media thickness in metabolic syndrome cohorts, providing clinical corroboration of the endotoxemia–atherosclerosis link [101,102].

Pancreatic β-cells represent a further downstream target of the endotoxemia–inflammation axis. Chronic exposure to IL-1β, generated through NLRP3 inflammasome activation in both islet-resident macrophages and adipose tissue, induces β-cell endoplasmic reticulum (ER) stress through activation of the PERK/eIF2α/CHOP pro-apoptotic arm of the unfolded protein response and dysregulation of IRE1α/XBP1-mediated adaptive signaling, leading to mitochondrial dysfunction and apoptosis that progressively reduce insulin secretory capacity and drive the transition from insulin resistance to overt T2DM [103,104,105]. This pancreatic dimension closes a pathogenic loop: barrier dysfunction generates the endotoxin signal that initiates adipose and hepatic insulin resistance, while the resulting hyperglycemia and metabolic stress further compromise barrier integrity, perpetuating both endotoxemia and β-cell decline in a self-amplifying cycle [6,88,106].

## 7. Rationale for Natural Product-Based Approaches

Current pharmacological management of cardiometabolic disease relies on agents that target downstream metabolic endpoints, including metformin for hepatic gluconeogenesis, statins for cholesterol biosynthesis, and SGLT2 inhibitors for renal glucose reabsorption, none of which were designed to address the upstream intestinal permeability defect and endotoxin translocation that initiate and sustain systemic inflammation [107,108]. This therapeutic gap is compounded by the absence of any approved drug whose primary indication is the restoration of gut barrier integrity in the context of metabolic disease, leaving the most proximal node in the endotoxemia–inflammation–insulin resistance cascade without a targeted intervention.

Natural bioactive compounds offer a conceptually distinct therapeutic approach because many of them accumulate at pharmacologically relevant concentrations within the intestinal lumen and mucosa irrespective of their systemic bioavailability [9]. Polyphenols, alkaloids, and dietary polysaccharides that undergo limited intestinal absorption retain biologically meaningful mucosal concentrations; this preferential luminal enrichment positions them precisely at the anatomical site where barrier reinforcement, LPS detoxification, and microbiota modulation exert maximum upstream benefit [10]. Mechanistically, several of these compounds simultaneously promote tight-junction reassembly, suppress TLR4/NF-κB signaling, restore the mucus layer, and enrich SCFA-producing commensals, yielding a convergent multi-target profile that is not currently matched by approved pharmacotherapies specifically directed at gut barrier restoration [22,109,110].

While traditional dietary and medicinal use provides a baseline safety signal for many of these compounds, formal dose-escalation safety studies remain necessary for therapeutic applications, as dose-dependent adverse effects have been documented for several agents, including gastrointestinal symptoms with berberine and hepatotoxicity with concentrated EGCG extracts [111]. The scalability of extraction and standardization processes, and the potential for integration into dietary patterns rather than pharmacological regimens, further support the rationale for investigating natural compounds as gut-targeted strategies in cardiometabolic disease [22,112]. The following section evaluates selected candidates that have accumulated the most robust mechanistic and translational evidence for barrier protection and endotoxemia attenuation.

## 8. Selected Natural Compounds

### 8.1. Polyphenols

#### 8.1.1. Curcumin

Curcumin, a polyphenolic compound and the biologically dominant constituent of the curcuminoid complex, is derived from the rhizome of *Curcuma longa* L. and has been used in Ayurvedic and Chinese medicinal traditions for millennia [113]. Commercial extracts are standardized to a minimum of 95% total curcuminoids, with curcumin comprising approximately 77% of the mixture; the remaining demethoxycurcumin and bisdemethoxycurcumin analogs exhibit higher bioavailability but lower anti-inflammatory and antioxidant capacity [113,114]. This breadth of activity makes curcumin a particularly relevant candidate for conditions driven by gut-derived endotoxemia and systemic metabolic inflammation [115].

At the mechanistic level, curcumin reinforces gut barrier integrity simultaneously at multiple levels. Pre-epithelially, it upregulates intestinal alkaline phosphatase, detoxifying LPS lipid A before it reaches the epithelial surface [115]. At the tight junction level, it prevents LPS- and IL-1β-driven p38 MAPK activation, blocking MLCK-mediated phosphorylation of ZO-1, claudin-1, and occludin and preserving the paracellular seal [116]. Upstream, it disrupts TLR4 dimerization and blocks MyD88/NF-κB-driven transcription of TNF-α, IL-6, and IL-1β, attenuating the inflammation-permeability amplification cycle at its innate immune origin [117].

These mechanistic observations are corroborated by convergent preclinical evidence. In STZ/HFD-induced diabetic rats, curcumin at 200 mg/kg/day for ten weeks significantly reduced serum LPS and TNF-α, suppressed intestinal TLR4/NF-κB, restored ultrastructural tight junction integrity, and reversed dysbiosis toward a reduced Gram-negative LPS load [118]. In LDLR-/- mice, curcumin and the LPS-neutralizing antibiotic polymyxin B produced equivalent reductions in plasma LPS and plaque burden, directly linking gut-derived endotoxin reduction to systemic vascular protection [115].

Beyond this preclinical evidence, clinical data provide supportive but contextually important findings. A meta-analysis of 26 RCTs demonstrated significant reductions in fasting glucose, HbA1c, HOMA-IR, triglycerides, LDL-C, and total cholesterol, with lipid improvements most pronounced at doses exceeding 500 mg/day [119]. A further meta-analysis in metabolic syndrome patients confirmed reductions in CRP, TNF-α, body weight, and BMI, strongest with bioavailability-enhanced formulations [120]. These findings are consistent with curcumin’s experimentally established gut barrier action [115].

These clinical findings must, however, be interpreted in light of significant methodological limitations. Substantial heterogeneity across RCT meta-analyses is driven by variation in formulation type, dose, duration, and participant phenotype [121]. Systemic bioavailability remains limited due to rapid phase II conjugation and colonic catabolism, though this does not preclude pharmacological relevance at the mucosal level, where luminal concentrations remain therapeutically meaningful [114,115,122]. To address both concerns, future trials should incorporate plasma LPS and permeability markers as primary endpoints and standardize bioavailability-enhanced formulations [114].

Taken together, curcumin’s mechanistic depth, convergent preclinical evidence, and supportive clinical data position it as a physiologically plausible gut-targeted intervention in cardiometabolic disease, though definitive efficacy claims await trials designed to directly test the gut barrier axis as a primary endpoint.

#### 8.1.2. Resveratrol

Resveratrol is a stilbenoid phytoalexin polyphenol found in grape skin and seeds, peanuts, blueberries, and Polygonum cuspidatum root, the primary source for commercial production [123,124,125,126]. Initially studied in the context of the “French paradox”, the epidemiological observation linking moderate red wine consumption to unexpectedly low cardiovascular disease rates in populations consuming high-fat diets, it has since been documented to exert antioxidant, anti-inflammatory, antidiabetic, antitumor, and neuroprotective activities, with particular relevance to cardiometabolic disease through preservation of intestinal barrier integrity, suppression of LPS translocation, and microbiota modulation away from endotoxin-producing Gram-negative species [123,127,128,129].

At the mechanistic level, resveratrol protects the intestinal epithelial barrier across multiple structural layers. It restores expression and membrane anchoring of ZO-1, ZO-2, occludin, claudin-1, and claudin-2, proteins whose disorganization under high-fat dietary conditions or LPS challenge allows Gram-negative bacteria to breach the epithelium, and upregulates mucin-2 and trefoil factor 3 in goblet cells, reinforcing the mucus barrier against luminal endotoxin [123,130]. At the signaling level, it inhibits LPS-driven TLR4/MyD88/NF-κB activation, suppressing IL-1β, IL-6, and TNF-α transcription and attenuating enterocyte apoptosis via Bax and Caspase-3 downregulation, while parallel SIRT1/AMPK activation drives CDX2-mediated TJ reassembly and IκB stabilization independently of TLR4 [130,131]. At the immune barrier level, resveratrol shifts mucosal T-cell balance toward CD4+ FOXP3+ regulatory T cells while reducing pro-inflammatory Th1 and Th17 populations in the lamina propria and mesenteric lymph nodes, preventing the inflammatory amplification loop [123].

These mechanistic observations are corroborated by convergent preclinical evidence. In HFD-fed mice, resveratrol restored tight junction proteins, reduced serum LPS and pro-inflammatory cytokines, and remodeled microbiota toward barrier-protective species [132]. In db/db diabetic mice, it normalized circulating LPS, TNF-α, IFN-γ, and IL-6 alongside ZO-1 and claudin-1 restoration, with FMT experiments confirming microbiota remodeling alone reproduced these outcomes [129]. In a septic rat model, resveratrol pretreatment preserved villus morphology, restored goblet cell density, reduced D-lactic acid and I-FABP, and reversed TLR4/MyD88/NF-κB upregulation, collectively demonstrating dual barrier reinforcement and microbiota-mediated endotoxin reduction [131].

Beyond this preclinical evidence, clinical data provide supportive but contextually important findings. A meta-analysis of 17 multinational RCTs demonstrated significant HOMA-IR reductions, with the most pronounced lipid improvements in the metabolic syndrome subgroup [133]. A larger 40-RCT meta-analysis confirmed reductions in HOMA-IR, total cholesterol, triglycerides, LDL-cholesterol, systolic blood pressure, TNF-α, IL-6, and hs-CRP, while body weight, BMI, HDL-cholesterol, and liver enzymes remained unchanged [127]. In patients with coronary artery disease and type 2 diabetes, resveratrol additionally improved HbA1c, systolic blood pressure, LDL-C, and flow-mediated dilation [124,134].

These clinical findings must, however, be interpreted in light of substantial methodological limitations. Paradoxically, the intestinal epithelium resveratrol protects is also the primary site of its inactivation, glucuronidation and sulfation in enterocytes and hepatocytes leave only a small fraction systemically available, while colonic microbial reductases convert the remainder into dihydroresveratrol and lunularin, metabolites that do not replicate the parent compound’s barrier-protective effects [123,128,130]. The 40-RCT meta-analysis reported substantial heterogeneity driven by variation in formulation, dose, duration, and phenotype, with subgroup analyses identifying a narrow glycemic window of four weeks or less at 100–500 mg/day consistent with compensatory SIRT1/AMPK downregulation [127]. Critically, since microbiota composition determines both resveratrol’s metabolite profile and remodeling capacity, the absence of baseline microbiota stratification across all published RCTs represents a critical uncontrolled variable [129,133].

Future trials incorporating microbiota stratification, standardized formulations, and adaptive dose-duration designs are needed to resolve whether resveratrol’s preclinical coherence translates into consistent cardiometabolic benefit.

#### 8.1.3. Quercetin

Quercetin is a flavonol belonging to the flavonoid subclass of plant polyphenols, found abundantly in red onions, capers, broccoli, apples, and berries, with onion-derived glucosides demonstrating the highest intestinal absorption efficiency among all dietary forms [135]. Given the breadth of its anti-inflammatory, antidiabetic, and barrier-protective activities, quercetin represents a relevant candidate for pathologies driven by chronic metabolic endotoxemia [136].

At the mechanistic level, quercetin acts through a coordinated set of mechanisms spanning tight junction assembly, innate immune regulation, and epithelial cell survival. At the tight junction level, it promotes the assembly of ZO-2, occludin, and claudin-1 through direct inhibition of PKCδ, preventing LPS-driven tight junction disorganization and the consequent increase in paracellular endotoxin flux [137]. At the innate immune signaling level, quercetin suppresses TLR4/NF-κB-driven transcription of TNF-α, IL-6, and IL-1β under LPS challenge, attenuating the cytokine-driven amplification of barrier permeability [138]. Additionally, quercetin inhibits NLRP3 inflammasome activation and gasdermin D-mediated pyroptotic cell death in intestinal epithelial cells, a mechanistic contribution to barrier preservation distinct from both curcumin and resveratrol [139].

These mechanistic observations are corroborated by convergent preclinical evidence. In db/db diabetic mice, quercetin reduced circulating D-lactic acid and serum LPS while restoring tight junction protein expression and improving insulin resistance [140]. The causal contribution of microbiota remodeling was confirmed when antibiotic-induced depletion abolished these barrier-protective effects entirely, with microbiota-driven isovanillic acid production identified as a key downstream mediator [141].

Beyond this preclinical evidence, clinical data provide supportive but contextually important findings. A meta-analysis of 7 RCTs demonstrated significant reductions in systolic and diastolic blood pressure, most consistently at doses exceeding 500 mg/day, and an umbrella review of RCT meta-analyses independently confirmed reductions in systolic blood pressure and fasting insulin across multiple pooled datasets [142,143]. Quercetin additionally attenuates ox-LDL oxidation and vascular smooth muscle cell proliferation, addressing two mechanistically central drivers of atherosclerotic plaque progression, and improves endothelial NO bioavailability and suppresses endothelial NF-κB activation, with gut-derived LPS reduction increasingly implicated as an upstream initiating event for these vascular benefits [144,145].

These clinical findings must, however, be interpreted in light of significant methodological limitations. Variable oral bioavailability, determined by glycoside form and downstream phase II glucuronidation and sulfation, produces inconsistent systemic exposure across individuals and study populations [135]. The causal role of microbiota composition in governing quercetin’s barrier efficacy, combined with the absence of microbiota stratification in all published RCTs, represents a critical and unresolved source of response variability [141].

#### 8.1.4. EGCG/Green Tea Catechins

Green tea is produced by steaming unfermented leaves to preserve the native catechin pool, of which EGCG constitutes approximately 50% of total polyphenols, the highest fraction among eight identified catechins [146,147]. More hydroxylated than any other green tea catechin, EGCG exhibits superior antioxidant potency and broad-spectrum binding affinity across disease-relevant targets, and is widely recognised for anti-inflammatory, antioxidant, antidiabetic, antihypertensive, and barrier-protective effects [146,147,148,149].

At the mechanistic level, what distinguishes EGCG is its capacity to act on gut barrier integrity through at least four independent but converging routes. EGCG acts on tight junctions by preventing claudin-1 and -5 delocalization in the colon while simultaneously upregulating the sealing claudin-4 and suppressing the leak-forming claudin-2 in the small intestine, collectively reducing macromolecular permeability. It counteracts apoptosis-dependent barrier leak driven by pro-inflammatory cytokines TNF-α and IFN-γ through caspase-3 cleavage inhibition [150]. By engaging the 67 kDa laminin receptor, EGCG disrupts the feedforward loop in which TLR4 activation by LPS triggers NF-κB and MAPK cascades that perpetuate barrier deterioration [147,151]. At the microbiota level, EGCG selectively expands barrier-protective commensal species while suppressing LPS-generating Gram-negative taxa; the resulting SCFA enrichment engages AhR–IL-22 and Nrf2/HO-1 axes to reinforce tight junction transcription, with Nrf2/HO-1 activation independently confirmed in intestinal epithelial models [152,153].

These mechanistic observations are first corroborated at the preclinical level. In HFD-fed rats, EGCG reduced circulating LPS, TNF-α, and IL-6 via TLR4/NF-κB suppression with restored microbial diversity, effects confirmed microbiota-dependent [152]. In db/db mice it improved glucose tolerance comparably to rosiglitazone, and in spontaneously hypertensive rats restored NO bioavailability through PI3K/Akt/eNOS activation [146,148].

Translating these findings to humans remains limited but directionally consistent. A green tea extract trial in metabolic syndrome patients demonstrated microbiota shifts paralleling reduced LPS-related metabolic output [154]. Moreover, a randomized double-blind placebo-controlled crossover trial confirmed reductions in serum endotoxin, intestinal inflammation markers, small-intestinal permeability, and fasting glucose [155]. These results, while encouraging, derive from a small number of trials with heterogeneous designs, and broader clinical validation remains warranted.

Beyond this evidence base, cardiometabolic effects of EGCG are among the most consistently documented of any dietary polyphenol. A meta-analysis of 11 RCTs confirmed reductions in waist circumference and triglycerides with increased HDL-C [156]. Furthermore, a separate meta-analysis showed significant SBP and DBP reductions pharmacologically coherent with the PI3K/Akt/eNOS mechanism [148,157].

These findings must be interpreted in light of significant translational limitations. COMT-mediated metabolism limits systemic bioavailability, partially offset by luminal retention and colonic generation of bioactive ring-fission metabolites [149,152,158]. Hepatotoxicity at doses exceeding 800 mg/day EGCG represents a dose-ceiling safety concern absent from the other polyphenols reviewed here, restricting the viable therapeutic window [147,158].

#### 8.1.5. Anthocyanins

Anthocyanins are water-soluble polyphenolic pigments of the flavonoid class, biosynthesised through the phenylpropanoid pathway and concentrated in the red, purple, and blue tissues of berries, grapes, red cabbage, purple corn, and eggplant. The six principal aglycones differ in B-ring hydroxylation and methoxylation patterns governing both pigmentation and bioactivity, and are consumed predominantly as glycosidic conjugates requiring intestinal hydrolysis prior to absorption [159].

At the mechanistic level, anthocyanins upregulate occludin, ZO-1, claudin-1, and MUC2 mucin in the intestinal epithelium while elevating plasma GLP-2, collectively reducing paracellular permeability and LPS translocation [160,161]. These effects are coupled to NOX1/NOX4 suppression, NF-κB p65 and ERK1/2 attenuation, and NOS2 inhibition, identifying redox and inflammatory signal crosstalk as the primary nodes through which anthocyanins preserve epithelial integrity under metabolic stress [160,162,163]. Anthocyanin supplementation additionally corrects HFD-induced dysbiosis while colonic catabolism yields phenolic metabolites that independently suppress NF-κB-mediated epithelial permeabilisation [161,164].

These mechanistic observations are first corroborated at the preclinical level. In HFD-fed C57BL/6J mice, anthocyanin supplementation fully prevented FITC-dextran flux and plasma endotoxin rise, restoring ileal occludin and claudin-1 to control levels [160]. In vitro, berry anthocyanins prevented TNFα-induced Caco-2 permeabilisation at concentrations ten times lower than those detected in human ileal tissue after 300 g raspberry consumption, while Lycium ruthenicum anthocyanins expanded Akkermansia and elevated butyrate sufficient to restore tight junction expression [160,165,166].

Translating these findings to humans remains limited but directionally consistent. A meta-analysis of 32 RCTs demonstrated significant reductions in fasting glucose, HbA1c, total cholesterol, and LDL-C [167]. Another meta-analysis of 29 RCTs found that 100 mg/day was sufficient to improve HDL-C and waist circumference [168]. Additionally, a nine-cohort meta-analysis found a significant inverse association between anthocyanin intake and CVD mortality, corroborated by the Iowa Women’s Health Study and an NHANES-linked cohort showing lower all-cause and heart disease-related mortality in the highest intake quartile [169,170]. These results, while encouraging, derive from trials with heterogeneous designs, and broader clinical validation remains warranted.

These clinical findings must, however, be interpreted in light of significant translational limitations. Oral bioavailability remains consistently low due to flavylium cation instability, food matrix interactions, and extensive colonic catabolism, while inter-individual variability in UGT, SULT, and COMT expression compounded by microbiota differences generates substantial ADME heterogeneity across trials [164,171,172]. The use of heterogeneous anthocyanin sources without standardised quantification further precludes reliable dose–response modelling [167].

### 8.2. Alkaloids

#### Berberine

Berberine is an isoquinoline alkaloid concentrated in the root and bark tissues of Berberis, Coptis, and Phellodendron species, isolated through methanol extraction and acid–base purification from Coptis chinensis rhizome [173,174]. Classified as a plant alkaloid rather than a polyphenol, berberine targets metabolic disease through a distinct signaling repertoire, with AMPK, PCSK9, LDLR, and gut microbial bile acid metabolism as its most thoroughly validated molecular nodes [175].

At the mechanistic level, berberine protects gut barrier integrity through a mechanistically layered sequence. It restores ZO-1, occludin, and claudin-1 expression while preventing myosin light chain phosphorylation, counteracting MLCK-dependent cytoskeletal retraction that opens the paracellular leak pathway under inflammatory conditions [174,176]. Berberine additionally stimulates mucin gene transcription, expands Akkermansia muciniphila, and suppresses LPS-producing Gram-negative taxa while enriching SCFA-producing commensals [173,177,178]. All four mechanisms converge on NF-κB suppression reinforced by AMPK activation via a lysosomal AXIN1-dependent pathway, reducing inflammatory signalling downstream of TLR4 [179,180,181].

These mechanistic observations are first corroborated at the preclinical level. In HFD-induced obese mice, berberine reduced circulating LPS, restored tight junction proteins, and attenuated hepatic inflammation and insulin resistance in a barrier-dependent manner [182]. In streptozotocin-induced diabetic rats, it restored occludin and ZO-1 in colonic mucosa while simultaneously improving glycaemic control and intestinal morphology [174].

Translating these findings to humans remains limited but directionally consistent. Human data confirm microbiota shifts toward reduced LPS-producing taxa alongside improvements in glucose and lipid profiles, with a landmark RCT showing berberine matched metformin on glycaemic outcomes while additionally lowering cholesterol and triglycerides [183,184]. Meta-analyses further confirm HDL-C improvement and significant reductions in TG, FPG, and waist circumference, with durations of ≤90 days producing the strongest lipid responses [185,186]. However, these results derive from a limited number of trials and broader clinical validation remains warranted.

These clinical findings must, however, be interpreted in light of significant translational limitations. Oral bioavailability below 1%, driven by P-glycoprotein efflux and first-pass metabolism, is partially resolved by microbial conversion to more bioavailable forms, though CYP3A4 inhibition introduces drug interaction risk and gastrointestinal adverse effects above 1.5 g/day remain the primary barrier to standardisation [173,185,187].

### 8.3. Omega-3 Polyunsaturated Fatty Acids

Omega-3 polyunsaturated fatty acids (PUFAs) are long-chain fatty acids defined by a double bond at the third carbon from the methyl terminus, comprising three clinically relevant forms: α-linolenic acid (ALA), eicosapentaenoic acid (EPA), and docosahexaenoic acid (DHA) [188]. ALA is found in flaxseed, chia, and walnuts, while EPA and DHA derive primarily from oily marine fish and microalgal sources, with phospholipid-bound forms in krill and algal oil demonstrating potentially superior bioavailability and tissue incorporation [189,190].

At the mechanistic level, EPA and DHA incorporate into colonocyte phospholipid membranes, stabilizing lipid raft microdomains that anchor ZO-1, occludin, and claudin-containing tight junction complexes [191,192]. As GPR120 ligands, they suppress TLR4/NF-κB signaling via β-arrestin-2/TAB1–TAK1 blockade and stimulate intestinal alkaline phosphatase to detoxify luminal LPS prior to epithelial contact, while EPA additionally displaces arachidonic acid from membrane phospholipids, shifting synthesis toward pro-resolving resolvins and protectins [191,192,193,194]. Moreover, n-3 PUFA-driven expansion of Bifidobacterium and Roseburia with suppression of LPS-producing Desulfovibrio and E. coli further reduces the luminal endotoxin pool [193].

These mechanistic observations translate into a coherent body of clinical evidence. Plasma DHA levels were inversely associated with LBP and fecal zonulin in a post hoc exploratory analysis of the randomized controlled LIBRE trial using validated permeability biomarkers [191]. Moreover, n-3 PUFA supplementation consistently improved ALT/AST and serum lipid profiles in patients with NAFLD across multiple clinical trials, though effects on hepatic steatosis remain inconclusive [195]. The FFAME endotoxemia challenge RCT demonstrated that 3600 mg/day EPA+DHA attenuated LPS-induced fever with a consistent pattern across all 9 inflammatory markers, though no individual cytokine beyond fever reached statistical significance; 900 mg/day produced no detectable effect, establishing a clear therapeutic dose threshold [196]. The REDUCE-IT trial demonstrated that 4 g/day icosapent ethyl reduced MACE by 25% in statin-treated patients with hypertriglyceridemia [197]. Meta-analysis of 38 RCTs confirmed EPA monotherapy reduces cardiovascular mortality more effectively than combined EPA+DHA, suggesting EPA-specific mechanisms confer incremental benefit [198]. Collectively, this convergent mechanistic and clinical profile positions n-3 PUFAs among the most comprehensively evidenced natural compounds targeting the gut–vascular inflammatory axis in cardiometabolic disease [192,199,200].

These clinical findings must, however, be interpreted in light of significant translational limitations. Commercially available supplement doses likely fall below the threshold for meaningful gut barrier benefit [196]. Additionally, marine n-3 supplementation was associated with a 25% increased risk of atrial fibrillation across seven RCTs in a dose-dependent manner [201]. The REDUCE-IT trial’s mineral oil placebo, which may have elevated LDL-C and CRP in the control arm, remains a major methodological controversy potentially inflating the apparent MACE benefit, and no outcomes trial has incorporated LPS, LBP, or zonulin as endpoints, leaving the gut barrier pathway unvalidated in human CVD [191,202].

### 8.4. Natural Polysaccharides and Oligosaccharides

Dietary polysaccharides represent a structurally diverse class of natural compounds whose gut barrier-protective and endotoxemia-attenuating properties are mechanistically convergent despite distinct chemical origins. Pectins are acidic heteropolysaccharides extracted from citrus peel and apple pomace, studied for their prebiotic and barrier-modulatory properties [203,204]. Inulin-type fructans are fructose polymers from chicory root and Jerusalem artichoke, established as selective prebiotics supporting beneficial microbiota and intestinal adaptation [205]. Cereal β-glucans, the most clinically validated subclass milled from oat and barley bran, carry FDA/EFSA-approved cholesterol-lowering activity and barrier-protective effects scaling with molecular weight and viscosity [206,207]. Fucoidan, alginate, and laminarin are sulfated polysaccharides from brown seaweeds whose polyuronic architecture and degree of sulfation confer distinct immunomodulatory and prebiotic properties [208,209].

Mechanistically, all four classes converge on colonic fermentation to butyrate and propionate, which activate GPR43 and GPR109a on colonocytes to upregulate ZO-1, claudin-1/4, and occludin while suppressing NF-κB-driven cytokines, thereby closing the paracellular route through which luminal LPS enters systemic circulation [210,211]. Beyond this shared pathway, each class contributes a structurally determined additional mechanism: pectin restores goblet cell MUC2 secretion to rebuild the mucus layer that LPS must breach before reaching the epithelium [212]; inulin-type fructans inhibit TLR4/MyD88/NF-κB p65 signaling to prevent cytokine amplification while GLP-2-mediated defensin secretion reduces luminal Gram-negative pathobionts—the primary source of LPS [213,214]; β-glucans physically trap LPS within viscous intraluminal gels and shift macrophage signaling toward tolerogenic responses via Dectin-1, reducing the inflammatory consequence of any LPS that does translocate [215,216]; and fucoidan competitively blocks LPS binding to the TLR4/MD-2 complex at the receptor level, while alginate oligosaccharides activate PI3K/Akt to reinforce occludin anchoring under inflammatory stress [209,217]. Through these converging mechanisms, all four classes attenuate the LPS–TLR4 axis driving adipose inflammation, hepatic steatosis, and insulin resistance [199,200].

Preclinical models provide proof-of-concept across all four classes. Apple pectin reduced plasma LPS while upregulating claudin-1, intestinal alkaline phosphatase, and Bacteroidetes abundance, demonstrating simultaneous barrier repair and luminal LPS source reduction [218]. Oat β-glucan upregulated ZO-1/occludin and suppressed inflammatory cytokines in aging mice, prevented MASLD fibrosis through microbiota modulation, and preserved barrier integrity via Lachnospiraceae enrichment in colorectal cancer models [216,219,220]. Fucoidan upregulated claudin-1, occludin, ZO-1, and sIgA in DSS colitis models, while alginate increased Firmicutes abundance and plasma LPS clearance in metabolic disease models [209,217].

Clinical translation is most robust for inulin-type fructans and β-glucans, though trial heterogeneity and small sample sizes limit generalizability. An RCT of 10 g/day inulin in women with type 2 diabetes achieved significant reductions in plasma LPS and hsCRP, providing direct evidence that inulin-driven endotoxemia attenuation reduces cardiometabolic inflammation [221]. A subsequent inulin and FOS RCT confirmed degree-of-polymerization-dependent microbiota targeting, with FOS enriching Faecalibacterium prausnitzii and inulin boosting Bifidobacterium, both correlating with improved glycemic control [214]. Oat and barley β-glucans carry FDA- and EFSA-approved health claims for LDL cholesterol reduction via bile acid sequestration, though direct clinical evidence for barrier endpoints in humans remains limited for this class [207].

In this context, the importance of dietary polysaccharides lies not in their structural diversity, but in their functional convergence at the host–microbiome interface. Their shared ability to reinforce gut barrier integrity and suppress endotoxin translocation identifies gut permeability as a central, modifiable node in cardiometabolic disease pathogenesis. Targeting this upstream mechanism through polysaccharide-rich dietary patterns may represent a shift from downstream disease management toward primary control of gut-derived inflammation.

**Figure 2:**
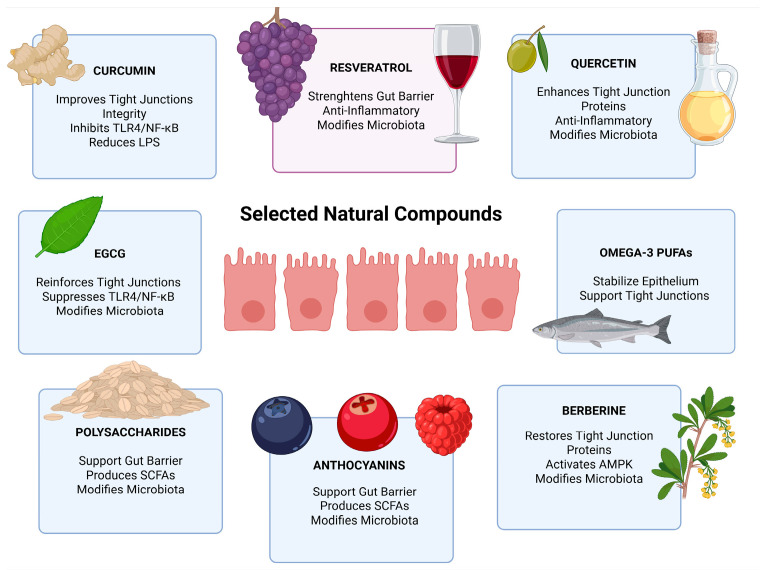
Natural compounds and compound classes targeting intestinal barrier integrity and metabolic endotoxemia. The central schematic depicts intestinal epithelial cells with an apical brush border. Curcumin reinforces tight junction integrity, inhibits TLR4/NF-κB signaling, and reduces lipopolysaccharide (LPS) burden. Resveratrol strengthens gut barrier function, exerts anti-inflammatory effects, and modifies gut microbiota composition. Quercetin enhances tight junction protein expression, suppresses inflammatory signaling, and modulates the microbiota. EGCG supports tight junction integrity, attenuates TLR4/NF-κB-mediated inflammation, and influences microbiota-related barrier effects. Dietary polysaccharides support barrier function primarily through microbiota modulation, short-chain fatty acid (SCFA) production, and luminal LPS trapping. Anthocyanins support barrier integrity, promote SCFA production, and modify microbial composition. Berberine restores tight junction proteins, activates AMP-activated protein kinase (AMPK)-dependent pathways, and modulates the microbiota. Omega-3 polyunsaturated fatty acids (PUFAs) stabilize epithelial membrane and tight junction complexes, attenuate LPS–TLR4 signaling, and generate pro-resolving lipid mediators. Abbreviations: AMPK, AMP-activated protein kinase; EGCG, epigallocatechin gallate; LPS, lipopolysaccharide; NF-κB, nuclear factor κB; PUFAs, polyunsaturated fatty acids; SCFAs, short-chain fatty acids; TLR4, Toll-like receptor 4. Created in BioRender. Kumric, M. (2026) https://BioRender.com/vjlm3cd (accessed on 21 May 2026).

This conceptual framework must, however, be interpreted in light of persistent translational challenges. Bioactivity remains highly dependent on fine structural features that are inconsistently characterized across studies, limiting comparability and reproducibility. Moving forward, progress will require standardized structural reporting and adequately powered randomized controlled trials designed to capture clinically meaningful cardiometabolic outcomes rather than surrogate endpoints. As illustrated in Figure 2, several natural compounds—including curcumin, resveratrol, quercetin, polysaccharides, anthocyanins, and berberine—exert protective effects on gut barrier function through mechanisms such as tight junction enhancement, anti-inflammatory signaling, SCFA production, and microbiota modulation.

## 9. Comparative Overview

Despite belonging to distinct chemical classes, including polyphenols, isoquinoline alkaloids, polyunsaturated fatty acids, and complex carbohydrates, the compounds reviewed above appear to converge on several partially overlapping mechanisms at the gut barrier. Across preclinical and, to a lesser extent, clinical studies, many of these compounds have been reported to improve tight junction protein expression, attenuate TLR4/NF-κB mediated inflammatory signaling, lower endotoxin burden, and modulate gut microbiota composition toward a less proinflammatory profile. Collectively, these findings suggest that the gut barrier and metabolic endotoxemia axis may represent a tractable therapeutic target for structurally diverse natural products.

Within this shared framework, mechanistic differentiation exists at the level of primary signaling targets and barrier-protective mechanisms. Curcumin uniquely upregulates intestinal alkaline phosphatase, providing pre-epithelial LPS detoxification that reduces the endotoxin load before it contacts the epithelium [115]. Berberine operates through AMPK-dependent MLCK inhibition and PCSK9 suppression, engaging metabolic pathways distinct from the polyphenol class [174,175,176]. Omega-3 PUFAs contribute membrane-level stabilization of tight junction complexes through phospholipid incorporation and generate pro-resolving lipid mediators (resolvins, protectins) that actively terminate inflammation rather than merely suppressing its initiation [192,194]. Anthocyanins uniquely engage NOX1/NOX4 redox signaling, while quercetin is distinguished by NLRP3 inflammasome inhibition and gasdermin D suppression [160,161,162]. Polysaccharides act predominantly through SCFA-mediated GPR43/GPR109a activation and physical intraluminal LPS trapping, mechanistic routes that operate upstream of the epithelial signaling events targeted by the other compound classes [210,211].

A common translational limitation shared across all reviewed compounds is poor or variable systemic bioavailability, driven by extensive phase II conjugation, P-glycoprotein efflux, and inter-individual variability in gut microbiota-mediated metabolism. This pharmacokinetic constraint is partially mitigated by the recognition that the primary site of action for barrier protection is the intestinal mucosa itself, where luminal and mucosal concentrations remain pharmacologically relevant regardless of systemic exposure. The absence of baseline microbiota stratification and the lack of standardized permeability and endotoxemia endpoints (LPS, LBP, zonulin, FITC-dextran) across clinical trials remain universal methodological gaps that preclude reliable dose–response modeling and cross-compound comparison [149,151]. Despite the methodological heterogeneity outlined above, consistent cardiometabolic signals emerge across compounds, as summarized in Table 1.

## 10. Translational Challenges

The mechanistic evidence reviewed above provides a coherent rationale for targeting the gut barrier–endotoxemia axis in cardiometabolic disease with natural bioactive compounds. Translating this rationale into clinical practice, however, requires resolution of several interrelated methodological, pharmacokinetic, and regulatory challenges that currently fragment the evidence base and prevent standardized therapeutic application.

The most fundamental challenge is the absence of validated, standardized clinical endpoints for intestinal permeability and metabolic endotoxemia. Existing RCTs overwhelmingly rely on downstream cardiometabolic surrogates—fasting glucose, HOMA-IR, lipid profiles, inflammatory markers—without capturing the upstream barrier mechanism that the intervention is hypothesized to modulate [222]. Plasma LPS measurement itself is technically demanding due to assay interference from lipoproteins and plasma proteins, while LBP and soluble CD14 reflect hepatic acute-phase responses rather than real-time endotoxin flux [223,224]. Zonulin, the most widely cited permeability biomarker, has been questioned on the basis of assay cross-reactivity with complement C3 and properdin, raising doubts about its specificity as a tight junction regulator in clinical samples [225,226]. This concern is directly relevant to the interpretation of several studies cited in preceding sections that employ zonulin as a primary endpoint. Until a consensus panel of permeability biomarkers—ideally combining functional assessments (lactulose/mannitol ratio, FITC-dextran), circulating damage markers (I-FABP, citrulline), and endotoxemia indicators (LPS, LBP, EndoCAb)—is adopted as co-primary or mandatory secondary endpoints, the mechanistic pathway linking natural compound administration to barrier restoration to cardiometabolic improvement remains clinically unvalidated [227].

Pharmacokinetic heterogeneity represents a second major obstacle. Oral bioavailability varies dramatically across compounds and within the same compound class depending on formulation, food matrix, and individual metabolic phenotype. Some of these compounds, particularly the polyphenolic agents, undergo extensive phase II conjugation in enterocytes and hepatocytes, resulting predominantly in glucuronidated, sulfated, or methylated metabolites [135]. This gap is compounded by the absence of pharmacokinetic-pharmacodynamic modeling that links mucosal drug concentrations-rather than plasma levels-to functional barrier outcomes.

Inter-individual variability in gut microbiota composition is a likely but largely uncontrolled source of response heterogeneity in published trials. Gut microbiota can modify the bioavailability and metabolite profile of natural compounds, thereby influencing their biological effects [228]. Berberine, for instance, is converted by intestinal microbes into dihydroberberine, whereas resveratrol is transformed into metabolites such as dihydroresveratrol and lunularin [229]. Similarly, part of quercetin’s barrier-protective activity appears to depend on microbiota-derived isovanillic acid [141]. The absence of baseline microbiota stratification means that trial populations likely contain both responders and non-responders whose differential outcomes are averaged into attenuated or null aggregate effects, artificially inflating heterogeneity statistics.

Source material standardization poses an additional practical barrier. Natural compounds are extracted from botanically variable raw materials whose phytochemical profiles depend on cultivar, geographic origin, harvest conditions, and processing methods [230,231]. Without standardized reference materials and mandatory compositional reporting in clinical trials, dose–response relationships cannot be reliably constructed.

## 11. Future Perspectives

Advancing the translational agenda for gut barrier-targeted natural compounds in cardiometabolic disease requires methodological innovation across several domains. Proof-of-concept clinical trials should adopt multi-compartment designs that simultaneously capture intestinal permeability (lactulose/mannitol, FITC-dextran), endotoxemia burden (LPS, LBP, EndoCAb), mucosal integrity markers (I-FABP, citrulline), and conventional cardiometabolic endpoints within the same patient cohort, enabling within-trial mediation analysis that can formally test whether barrier restoration mediates downstream metabolic improvement [232,233].

Baseline microbiota profiling by shotgun metagenomics, combined with pre-specified stratification by enterotype or by the abundance of key metabolizing taxa, could help identify responder subpopulations and support enrichment-oriented trial designs aimed at testing mechanistically plausible treatment effects [234,235]. Paired metabolomic analysis of luminal, portal, and systemic compartments could resolve the outstanding question of whether parent compounds or their microbial metabolites are the primary effectors of barrier protection, thereby guiding rational formulation strategies.

Combinatorial approaches warrant further investigation, as different classes of natural compounds act through partially complementary mechanisms affecting barrier function and inflammation. For example, some compounds modulate endotoxin-related signaling, others influence luminal interactions or microbiota-dependent processes, while additional agents target intracellular pathways such as inflammasome activation or AMPK signaling [236,237,238]. This mechanistic diversity supports the potential for additive or synergistic effects in multi-compound interventions. Experimental platforms such as organ-on-chip systems and gut-liver co-culture models may facilitate controlled preclinical evaluation of such strategies [239].

## 12. Conclusions

This narrative review supports the concept that the gut barrier–endotoxemia–inflammation axis represents a biologically plausible upstream contributor to cardiometabolic disease. Disruption of intestinal epithelial integrity may facilitate chronic translocation of LPS and related microbial products, thereby promoting TLR4/NF-κB-driven inflammatory signaling in adipose tissue, liver, vasculature, and pancreatic islets. These mechanisms provide a coherent framework linking gut barrier dysfunction with insulin resistance, dyslipidemia, hepatic steatosis, endothelial dysfunction, and other features of the cardiometabolic syndrome phenotype.

The natural bioactive compounds evaluated in this review—curcumin, resveratrol, quercetin, EGCG, berberine, anthocyanins, omega-3 PUFAs, and dietary polysaccharides-show mechanistic overlap in their potential to modulate tight junction integrity, inflammatory signaling, gut microbiota composition, and endotoxin-related pathways. Preclinical studies provide substantial support for barrier-protective and anti-inflammatory effects across several compound classes; however, this evidence remains model-dependent and cannot be directly equated with clinical efficacy. In humans, randomized trials and meta-analyses suggest potential cardiometabolic benefits for some agents and outcomes, but the consistency of these effects varies across compounds, populations, doses, formulations, intervention durations, and measured endpoints. Accordingly, the current clinical evidence should be interpreted as supportive but not yet definitive, particularly because many trials have not directly assessed intestinal permeability, circulating endotoxemia markers, or mediation through barrier restoration.

A central translational gap is therefore the limited availability of trials that simultaneously measure barrier function, endotoxemia, inflammatory signaling, and cardiometabolic outcomes using standardized and validated endpoints. Future studies should incorporate biomarker panels that may include lactulose/mannitol ratio, plasma LBP, sCD14, EndoCAb, I-FABP, and citrulline, together with conventional metabolic and vascular outcomes. Baseline microbiota profiling and pre-specified responder stratification may also help clarify inter-individual variability and reduce the heterogeneity that currently limits interpretation. Finally, combinatorial interventions pairing mechanistically complementary compounds warrant investigation, but their additive or synergistic effects require formal testing in well-designed clinical trials. Overall, natural gut barrier-targeted compounds represent a promising and mechanistically grounded area of research, but their role in cardiometabolic disease should be considered investigational until more rigorous human evidence establishes whether barrier restoration translates into clinically meaningful benefit.

## Figures and Tables

**Table 1:** Concise summary of clinical evidence for selected natural products.

Compound	Clinical Evidence	Main Outcomes
Curcumin	RCT meta-analyses in metabolic disorders and MetS [119,120,121]	Improved lipids, glycemic indices, body weight/BMI, CRP and TNF-α
Resveratrol	RCT meta-analysis in MetS/obesity [133]	Improved HOMA-IR and selected cardiometabolic risk factors; effects heterogeneous
Quercetin	RCT meta-analysis and umbrella review [142,143]	Reduced SBP/DBP and fasting insulin
EGCG/green tea catechins	Direct gut-barrier RCT plus RCT meta-analyses [155,156,157]	Reduced endotoxin, permeability/inflammatory gut markers, fasting glucose, WC and TG; increased HDL-C
Berberine	Human T2D/microbiome RCTs and RCT meta-analyses [183,184,185,186]	Improved glucose control, lipid profile, WC/BMI and gut microbiome-related metabolic pathways
Anthocyanins	RCT meta-analyses and cohort/meta-analysis evidence [167,168,169,170]	Improved glycemic and lipid markers; inverse association with CVD/all-cause mortality
Omega-3 PUFAs	Barrier/endotoxemia RCTs, REDUCE-IT and RCT meta-analysis [191,196,197,198]	Improved barrier-related markers, attenuated LPS response, reduced TG and cardiovascular events
Polysaccharides/oligosaccharides	Inulin/FOS RCTs and β-glucan clinical evidence [207,214,221]	Reduced LPS and inflammatory markers; improved microbiota, glycemic markers and LDL-C

Abbreviations: BMI, body mass index; CRP, C-reactive protein; CVD, cardiovascular disease; DBP, diastolic blood pressure; FOS, fructooligosaccharides; HDL-C, high-density lipoprotein cholesterol; HOMA-IR, homeostatic model assessment of insulin resistance; LDL-C, low-density lipoprotein cholesterol; LPS, lipopolysaccharide; MetS, metabolic syndrome; RCT, randomized controlled trial; SBP, systolic blood pressure; TG, triglycerides; WC, waist circumference; T2D, type 2 diabeted; TNF-α, tumor necrosis factor alpha.

## Data Availability

No new data were created or analyzed in this study.

## Abbreviations

The following abbreviations are used in this manuscript:

T2DM	Type 2 Diabetes Mellitus
CVD	Cardiovascular Disease
NAFLD	Non-Alcoholic Fatty Liver Disease
LPS	Lipopolysaccharide
TLR4	Toll-Like Receptor 4
NF-κB	Nuclear Factor Kappa B
LBP	Lipopolysaccharide-Binding Protein
CD14	Cluster of Differentiation 14
MyD88	Myeloid Differentiation Primary Response 88
TRIF	TIR-domain-containing adapter-inducing interferon-β
NLRP3	NOD-like receptor family pyrin domain containing 3
TNF-α	Tumor Necrosis Factor Alpha
IL-1β/IL-6/IL-18	Interleukins
MAPK	Mitogen-Activated Protein Kinase
JNK/ERK/p38	MAPK podskupine
SCFA	Short-Chain Fatty Acids
EGCG	Epigallocatechin Gallate
PUFA	Polyunsaturated Fatty Acids
RCT	Randomized Controlled Trial
HOMA-IR	Homeostatic Model Assessment of Insulin Resistance
BMI	Body Mass Index
CRP	C-reactive Protein
GLUT4	Glucose Transporter Type 4
IRS-1	Insulin Receptor Substrate 1
AMPK	AMP-activated Protein Kinase
SIRT1	Sirtuin 1
ZO-1/ZO-2	Zonula Occludens proteins
MUC2	Mucin 2
MetS	Metabolic Syndrome
